# Impact of tumor size on the outcomes of hepatic resection for hepatocellular carcinoma: a retrospective study

**DOI:** 10.1186/s12893-023-02296-w

**Published:** 2024-01-03

**Authors:** Ahmed Shehta, Ahmed M. Elsabbagh, Mohamed Medhat, Ahmed Farouk, Ahmed Monier, Rami Said, Tarek Salah, Mohamed Elshobari, Amgad Fouad, Ahmed N. Elghawalby

**Affiliations:** Gastrointestinal Surgery Center, Department of Surgery, Faculty of Medicine, Mansoura University, Mansoura, Egypt

**Keywords:** Hepatocellular carcinoma, Liver resection, Tumor size, Survival

## Abstract

**Background:**

To evaluate the impact of tumor size on the perioperative and long-term outcomes of liver resection for hepatocellular carcinoma (HCC).

**Methods:**

We reviewed the patients’ data who underwent liver resection for HCC between November 2009 and 2019. Patients were divided into 3 groups according to the tumor size. Group I: HCC < 5 cm, Group II: HCC between 5 to 10 cm, and Group III: HCC ≥ 10 cm in size.

**Results:**

Three hundred fifteen patients were included in the current study. Lower platelets count was noted Groups I and II. Higher serum alpha-feto protein was noted in Group III. Higher incidence of multiple tumors, macroscopic portal vein invasion, nearby organ invasion and presence of porta-hepatis lymph nodes were found in Group III. More major liver resections were performed in Group III. Longer operation time, more blood loss and more transfusion requirements were found in Group III. Longer hospital stay and more postoperative morbidities were noted in Group III, especially posthepatectomy liver failure, and respiratory complications.

The median follow-up duration was 17 months (7–110 months). Mortality occurred in 100 patients (31.7%) and recurrence occurred in 147 patients (46.7%). There were no significant differences between the groups regarding recurrence free survival (Log Rank, *p* = 0.089) but not for overall survival (Log Rank, *p *= 0.001).

**Conclusion:**

HCC size is not a contraindication for liver resection. With proper selection, safe techniques and standardized care, adequate outcomes could be achieved.

## Background

Hepatocellular carcinoma (HCC) is one of the commonest malignancies worldwide, and the third most common cause of cancer-related death. It is the most common primary malignancy affecting the liver [1–3]. Liver resection and transplantation are the only available curative treatment lines for HCC patients. Owing to several limitations of liver transplantation, liver resection is considered the treatment of choice for HCC patients with solitary tumor and sufficient hepatic functional reserve. With the refinement of the surgical techniques, and advancement of perioperative care, the outcomes of liver resection have markedly improved in the recent years [4, 5]. On the other hand, local thermal ablation especially if combined with immunotherapy could provide effective control of the tumor with acceptable survival outcomes [6, 7].

Several factors had been identified to predict poor outcomes after curative liver resection for HCC as tumor size, number, presence of vascular invasion, tumor differentiation and serum tumor markers [8]. The modified American Joint Committee on Cancer (AJCC) tumor-lymph node-metastasis system (pTNM) includes tumor size, number, and vascular invasion in its tumor (T) classification. Therefore, tumor size should be considered as one of the most important predictive factors for tumor recurrence and patient survival [4, 9]. Also, it is known that the prognosis of patients undergoing curative resection for larger HCC is inferior to those with small HCC. This is attributed to the fact that larger HCCs are frequently associated with some adverse prognostic factors like the presence of microscopic vascular invasion [10, 11]. On the other hand, some studies from high-volume centers had shown the tumor size did not affect survival outcomes in patients who underwent liver resection for single HCC without vascular invasion [12, 13].

The current study was conducted to evaluate the impact of tumor size on the outcomes of liver resection for HCC among Egyptian patients, in area where hepatitis C virus (genotype 4) is the main predisposing factor for HCC development and evaluate the prognostic impact of tumor size on the long-term survival outcomes.

## Methods

### Study design

We retrospectively review the data of patients who underwent primary liver resection for HCC at Gastro-intestinal Surgery Center (GISC), Mansoura University, Egypt during the period between November 2009 and November 2019. Patients were divided into 3 groups according to the tumor size in the final pathological report. Group I included patients with tumors < 5 cm in size, Group II included patients with tumors between 5 to 10 cm in size, and Group III included patients with tumors ≥ 10 cm in size.

Patient data were retrieved from a prospectively maintained database for all patients undergoing liver resection. An informed consent was obtained from each patient prior to surgical intervention. The study was approved by the Institutional Review Board and Local Ethical Committee at the Faculty of Medicine, Mansoura University, Egypt (R.20.06.874). The current study methods were carried out in accordance with the Declaration of Helsinki.

### Preoperative evaluation

Preoperative workup included detailed clinical, laboratory, and radiological evaluation was described before [8, 14]. Generally, liver resection was applied for patients with preserved liver functions (i.e., sufficient future liver remnant), without signs of severe portal hypertension, without evidence of extrahepatic metastasis, and with American Society of Anesthesiologists (ASA) grade < III [15].

### Surgical procedure

The surgical procedure had been described elsewhere [8, 14]. The types of liver resection were defined according to Brisbane 2000 terminology [16]. Generally, parenchymal sparing liver resection was preferred for fear of postoperative liver dysfunction. Major liver resections were performed for patients with large tumors or tumors close to major hepatic vasculature if the future remnant liver is adequate (more than 40% of the total liver volume). Volumetric assessment was performed for selected patients requiring major liver resection with marginal liver functions. Otherwise, non-anatomical liver resections were more preferred. Liver parenchymatous transection was performed by combinations of clamp-crush method and ultrasonic devices. Intermittent Pringle’s maneuver was applied selectively during liver transection. Intraoperative ultrasonography (Canon, Xario 200, Japan) was utilized in some patients to check the resection margin and exclude presence of multifocal tumors [8, 14].

### Postoperative care and follow up

After surgery, patients were transferred to the intensive care unit or to the ward for monitoring. All patients underwent daily laboratory evaluation. Abdominal ultrasonography was performed routinely in all patients. Oral fluids were started once intestinal sounds are restored. Abdominal drains were removed when daily output was less than 100 cc with absence of any abdominal collections [8, 14].

After discharge, patients were followed-up in the outpatient clinic. Follow-up visit included physical examination, serum liver function tests, serum alpha fetoprotein, abdominal ultrasonography, and triphasic computed tomography when recurrence was suspected [8, 14].

### Definitions

Postoperative morbidity is defined as adverse events happening during the early postoperative period and is graded according to the Clavien-Dindo classification [17]. Postoperative liver dysfunction, biliary fistula and hemorrhage are defined according to the ISGLS definitions [18–20]. Early postoperative mortality was defined as mortality occurring during the first 90 postoperative days and was excluded from further survival analysis.

Overall survival (OS) was calculated from the day of surgery to the day of confirmed death or the last follow up visit. Disease-free survival (DFS) was calculated from the day of surgery to the day of confirmed tumor recurrence or the day of death or last follow up.

### Statistical analysis

Categorical variables were expressed as number (percentage), and continuous variables were expressed as median (range). Comparison between the three groups was done by chi-square or ANOVA test when appropriate and comparison between each two groups was done by pair-wise comparisons. Survival rates were calculated by Kaplan–Meier method, and comparison between groups was done by Log-Rank test.

Statistical analysis was performed using the SPSS 22 software (IBM, Chicago, IL, USA). A *p* value less than 0.05 was considered statistically significant.

## Results

During the period between November 2009 and November 2019, 315 patients with pathologically confirmed hepatocellular carcinoma underwent liver resection at Gastrointestinal Surgery Center, Mansoura University, Egypt.

Patients were divided into 3 groups according to the tumor size. Group I included patients with tumors < 5 cm in size (88 patients – 26.7%). Group II included patients with tumors between 5 to 10 cm in size (167 patients – 50.8%). Group III included patients with tumors ≥ 10 cm in size (60 patients – 18.2%).

### Demographic data

The demographic data of the study patients were summarized in Table 1. There were significant differences between the groups regarding clinical presentation, serum albumin, serum alanine aminotransferase, platelets count, serum alpha-feto protein, and hepatitis C virus antibodies. Higher incidence of accidentally discovered HCC was noted in Group I and II while more abdominal pain and masses were noted in Group III. Higher serum albumin and alanine aminotransferase were noted in Group I, while lower platelets counts was noted Groups I and II. Higher serum alpha-feto protein was noted in Group III.

**Table 1:** Demographic data of the study patients (*TACE* Trans-arterial chemo-embolization, *RFA* Radiofrequency ablation)

Variables	All (*N* = 315)	Group I (*N* = 88)	Group II (*N* = 167)	Group III (*N* = 60)	*P* value
Age (years)	60 (18–78)	60 (41–73)	60 (22–78)	60 (18–73)	0.679
Gender					0.739
Male	252 (80%)	72 (81.8%)	134 (80.2%)	46 (76.7%)	
Female	63 (20%)	16 (18.2%)	33 (19.8%)	14 (23.3%)	
Body mass index (kg/m^2^)	28.7 (17.3–42.7)	27.9 (20.8–39.6)	28.7 (17.3–39.7)	29.1 (21–42.7)	0.541
Previous abdominal operations	98 (31.1%)	30 (34.1%)	55 (32.9%)	13 (21.7%)	0.21
Previous TACE	20 (6.3%)	6 (6.8%)	11 (6.6%)	3 (5%)	0.89
Previous RFA	5 (1.6%)	1 (1.1%)	3 (1.8%)	1 (1.7%)	0.921
Complaint					0.023
Accidental	161 (51.1%)	47 (53.4%)	90 (53.9%)	24 (40%)	
Pain	152 (48.3%)	41 (46.6%)	77 (46.1%)	34 (56.7%)	
Mass	2 (0.6%)	0	0	2 (3.3%)	
Previous antiviral therapy	33 (10.5%)	11 (12.5%)	13 (7.8%)	9 (15%)	0.225
Albumin (g/dL)	3.9 (2.1–5.3)	4 (3–5)	3.9 (2.2–5.3)	3.8 (2.1–4.7)	0.012
Bilirubin (mg/dL)	0.7 (0.3–11.2)	0.7 (0.4–2)	0.7 (20–275)	0.7 (0.4–2)	0.829
Alanine aminotransferase (IU/L)	41 (20–280)	48.5 (20–182)	42 (20–275)	35.5 (20–280)	0.023
Aspartate aminotransferase (IU/L)	49 (20–240)	50 (20–190)	49 (20–218)	48.5 (20–240)	0.944
International normalized ratio	1 (1–2)	1 (1–1.4)	1 (1–2)	1 (1–1.4)	0.741
Platelets (× 10^3^/mL)	146 (34–433)	141.5 (34–319)	143 (43–381)	185 (55–433)	0.006
Creatinine (mg/dL)	0.8 (0.5–2.5)	0.8 (0.5–2.5)	0.8 (0.5–2.3)	0.8 (0.5–2.3)	0.359
Alpha feto-protein (ng/ml)	30.8 (1–3000)	19.1 (1–2000)	27.9 (1.5–3000)	1000 (1.5–2000)	0.007
Child–Pugh grade					0.247
A	308 (97.8%)	88 (100%)	162 (97%)	58 (96.7%)	
B	7 (2.2%)	0	5 (3%)	2 (3.3%)	
Model for end stage liver disease (MELD score)	7 (6–17)	7 (6–13)	7 (6–16)	7 (6–17)	0.654
Hepatitis C virus	291 (92.4%)	86 (97.7%)	155 (92.8%)	50 (83.3%)	0.005
Hepatitis B virus	3 (1%)	1 (1.1%)	1 (0.6%)	1 (1.7%)	0.749 s

Presentation: Group I vs II *p* = 0.941, Group I vs III *p* = 0.083, Group II vs III *p* = 0.016

Albumin: Group I vs II: *p* = 0.267, Group I vs III: *p* = 0.08, Group II vs III: *p* = 0.707

ALT: Group I vs II *p* = 0.815, Group I vs III *p* = 0.027, Group II vs III *p* = 0.069

Platelets: Group I vs II: *p* = 1, Group I vs III: *p* = 0.022, Group II vs III: *p* = 0.002

AFP: Group I vs II: *p* = 0.935, Group I vs III: *p* = 0.003, Group II vs III: *p* = 0.012

HCV: Group I vs II *p* = 0.148, Group I vs III *p* = 0.004, Group II vs III *p* = 0.042

### Radiological and Endoscopic data

Radiological and endoscopic data of the study patients were summarized in Table 2. There were significant differences between the groups regarding liver status, tumor site and macroscopic portal vein invasion. Higher incidence of liver cirrhosis was found in Group I and II. Higher incidence of macroscopic portal vein invasion was found in Group III.

**Table 2:** Radiologic and endoscopic data of the study patients (*GIT* Gastro-intestinal tract)

Variables	All (*N* = 315)	Group I (*N* = 88)	Group II (*N* = 167)	Group III (*N* = 60)	*P* value
Liver status					0.018
Cirrhosis	300 (95.2%)	86 (97.7%)	161 (96.4%)	53 (88.3%)	
Normal	15 (4.8%)	2 (2.3%)	6 (3.6%)	7 (11.7%)	
Number					0.2
Single	286 (90.8%)	84 (95.5%)	149 (89.2%)	53 (88.3%)	
Multiple	29 (9.2%)	4 (4.5%)	18 (10.8%)	7 (11.7%)	
Site					0.001
Right hemi-liver	31 (9.8%)	2 (2.3%)	13 (7.8%)	16 (26.7%)	
Left hemi-liver	14 (4.4%)	3 (3.4%)	6 (3.6%)	5 (8.3%)	
Left lateral section	38 (12.1%)	7 (8%)	24 (14.4%)	7 (11.7%)	
Segment IV	16 (5.1%)	6 (6.8%)	7 (4.2%)	3 (5%)	
Right anterior section	3 (1%)	0	2 (1.2%)	1 (1.7%)	
Right posterior section	14 (4.4%)	1 (1.1%)	8 (4.8%)	5 (8.3%)	
Central	4 (1.3%)	2 (2.3%)	2 (1.2%)	0	
Caudate lobe	6 (1.9%)	2 (2.3%)	4 (2.4%)	0	
Segment II	24 (7.6%)	12 (13.6%)	10 (6%)	2 (3.3%)	
Segment III	39 (12.4%)	18 (20.5%)	17 (10.2%)	4 (6.7%)	
Segment V	15 (4.8%)	6 (6.8%)	9 (5.4%)	0	
Segment VI	49 (15.6%)	13 (14.8%)	27 (16.2%)	9 (15%)	
Segment VII	26 (8.3%)	7 (8%)	17 (10.2%)	2 (3.3%)	
Segment VIII	16 (5.1%)	5 (5.7%)	11 (6.6%)	0	
Multi-site	20 (6.3%)	4 (4.5%)	10 (6%)	6 (10%)	
Macroscopic portal vein invasion	38 (12.1%)	6 (6.8%)	15 (9%)	17 (28.3%)	0.001
Porta hepatis lymph nodes	44 (14%)	11 (12.5%)	21 (12.6%)	12 (20%)	0.326
Upper GIT endoscopy	306 (97.1%)	84 (95.5%)	164 (98.2%)	58 (96.7%)	0.443
Endoscopy findings					0.129
Esophageal veins	54 (17.1%)	18 (20.5%)	30 (18%)	6 (10%)	
Gastric compression	1 (0.3%)	0	0	1 (1.7%)	

Liver status: Group I vs II *p* = 0.718, Group I vs III *p* = 0.031, Group II vs III *p* = 0.045

Tumor site: Group I vs II *p* = 0.181, Group I vs III *p* = 0.001, Group II vs III *p* = 0.008

Portal invasion: Group I vs II *p* = 0.638, Group I vs III *p* = 0.001, Group II vs III *p* = 0.001

### Operative data

Operative data of the study patients were summarized in Table 3. Open conversion occurred in 2 cases (1.2%) because of bleeding. There were significant differences between the groups regarding liver status, tumor site, tumor number, macroscopic vascular invasion, nearby organ invasion, presence of lymph nodes, liver resection extent, liver resection type, Pringle maneuver indication, total operation time, operative blood loss, and blood transfusion requirements.

**Table 3:** Operative data of the study patients

Variables	All (*N* = 315)	Group I (*N* = 88)	Group II (*N* = 167)	Group III (*N* = 60)	*P* value
Liver status					0.001
Cirrhosis	296 (94%)	86 (97.7%)	160 (95.8%)	50 (83.3%)	
Normal	19 (6%)	2 (2.3%)	7 (4.2%)	10 (16.7%)	
Site					0.04
Right hemi-liver	160 (50.8%)	35 (39.8%)	90 (53.9%)	35 (58.3%)	
Left hemi-liver	141 (44.8%)	51 (58%)	68 (40.7%)	22 (36.7%)	
Caudate lobe	6 (1.9%)	2 (2.3%)	4 (2.4%)	0	
Bilobar	8 (2.5%)	0	5 (3%)	3 (5%)	
Lesion site details					0.001
Right hemi-liver	27 (8.6%)	2 (2.3%)	10 (6%)	15 (25%)	
Left hemi-liver	11 (3.5%)	1 (1.1%)	6 (3.6%)	4 (6.7%)	
Left lateral section	56 (17.8%)	14 (15.9%)	32 (19.2%)	10 (16.7%)	
Segment IV	16 (5.1%)	7 (8%)	6 (3.6%)	3 (5%)	
Right anterior section	2 (0.6%)	0	2 (1.2%)	0	
Right posterior section	12 (3.8%)	1 (1.1%)	6 (3.6%)	5 (8.3%)	
Central	10 (3.2%)	4 (4.5%)	5 (3%)	1 (1.7%)	
Caudate lobe	6 (1.9%)	2 (2.3%)	4 (2.4%)	0	
Segment II	13 (4.1%)	7 (8%)	6 (3.6%)	0	
Segment III	35 (11.1%)	20 (22.7%)	13 (7.8%)	2 (3.3%)	
Segment V	14 (4.4%)	6 (6.8%)	8 (4.8%)	0	
Segment VI	45 (14.3%)	11 (12.5%)	28 (16.8%)	6 (10%)	
Segment VII	32 (10.2%)	10 (11.4%)	19 (11.4%)	3 (5%)	
Segment VIII	15 (4.8%)	3 (3.4%)	11 (6.6%)	1 (1.7%)	
Multi-site	20 (6.3%)	0	11 (6.6%)	9 (15%)	
Number					0.003
Single	290 (92.1%)	87 (98.9%)	153 (91.6%)	50 (83.3%)	
Multiple	25 (7.9%)	1 (1.1%)	14 (8.4%)	10 (16.7%)	
Vascular invasion	41 (13%)	6 (6.8%)	17 (10.2%)	18 (30%)	0.001
Biliary invasion	1 (0.3%)	0	1 (0.6%)	0	0.641
Nearby organ invasion	19 (6%)	3 (3.4%)	7 (4.2%)	9 (15%)	0.005
Lymph nodes	25 (7.9%)	5 (5.7%)	9 (5.4%)	11 (18.3%)	0.004
Intraoperative biopsies	16 (5.1%)	3 (3.4%)	8 (4.8%)	5 (8.3%)	0.395
Biopsy site					0.299
Suspicious liver nodule	7 (2.3%)	2 (2.3%)	3 (1.9%)	2 (3.3%)	
Safety margin	1 (0.3%)	0	1 (0.6%)	0	
Lymph nodes	8 (2.5%)	1 (1.1%)	4 (2.4%)	3 (5%)	
Biopsy result					0.239
HCC	5 (1.6%)	0	3 (1.8%)	2 (3.3%)	
High grade tumor	1 (0.3%)	1 (1.1%)	0	0	
Negative	10 (3.2%)	2 (2.3%)	5 (3%)	3 (5%)	
Surgery approach					0.312
Open	308 (97.8%)	85 (96.6%)	163 (97.6%)	60 (100%)	
Laparoscopic	5 (1.6%)	3 (3.4%)	2 (1.2%)	0	
Failed laparoscopic	2 (0.6%)	0	2 (1.2%)	0	
Liver resection extent					0.001
Minor	241 (76.5%)	82 (93.2%)	131 (78.4%)	28 (46.7%)	
Major	74 (23.5%)	6 (6.8%)	36 (21.6%)	32 (53.3%)	
Liver resection type					0.001
Tumorectomy	149 (47.3%)	56 (63.6%)	82 (49.1%)	11 (18.3%)	
Segmentectomy	6 (1.9%)	0	6 (3.6%)	0	
Left lateral sectionectomy	72 (22.9%)	22 (25%)	35 (21%)	15 (25%)	
Right anterior sectionectomy	1 (0.3%)	1 (1.1%)	0	0	
Right posterior sectionectomy	1 (0.3%)	0	1 (0.6%)	0	
Left hepatectomy	14 (4.4%)	1 (1.1%)	7 (4.2%)	6 (10%)	
Extended left hepatectomy	1 (0.3%)	0	1 (0.6%)	0	
Right hepatectomy	52 (16.5%)	3 (3.4%)	27 (16.2%)	22 (36.7%)	
Extended right hepatectomy	6 (1.9%)	2 (2.3%)	1 (0.6%)	3 (5%)	
Central hepatectomy	1 (0.3%)	1 (1.1%)	0	0	
Caudate lobectomy	6 (1.9%)	2 (2.3%)	4 (2.4%)	0	
Multiple resections	6 (1.9%)	0	3 (1.8%)	3 (5%)	
Associated portal thrombectomy	6 (1.9%)	0	3 (1.8%)	3 (5%)	0.091
Associated extrahepatic biliary resection	1 (0.3%)	0	1 (0.6%)	0	0.641
Pringle procedure use	46 (14.6%)	9 (10.2%)	26 (15.6%)	11 (18.3%)	0.342
Pringle indication					0.007
Elective	27 (8.6%)	7 (8%)	18 (10.8%)	2 (3.3%)	
Emergency	19 (6%)	2 (2.3%)	8 (4.8%)	9 (15%)	
Pringle duration (minutes)	17.5 (10–90)	15 (10–25)	17.5 (10–90)	20 (15–40)	0.904
Operation time (hours)	3 (1.2–7)	2.5 (1.2–7)	3 (1.2–6)	3.5 (1.5–6)	0.001
Blood loss (ml)	600 (50–6000)	400 (50–4000)	600 (50–6000)	1000 (100–6000)	0.001
Blood transfusion	152 (48.3%)	25 (28.4%)	84 (50.3%)	43 (71.7%)	0.001

Liver status: Group I vs II *p* = 0.723, Group I vs III *p* = 0.004, Group II vs III *p* = 0.003

Tumor site: Group I vs II *p* = 0.034, Group I vs III *p* = 0.009, Group II vs III *p* = 0.516

Tumor site details: Group I vs II *p* = 0.013, Group I vs III *p* = 0.001, Group II vs III *p* = 0.001

Number: Group I vs II *p* = 0.023, Group I vs III *p* = 0.001, Group II vs III *p* = 0.088

Vascular invasion: Group I vs II *p* = 0.492, Group I vs III *p* = 0.001, Group II vs III *p* = 0.001

Organ invasion: Group I vs II *p* = 1, Group I vs III *p* = 0.015, Group II vs III *p* = 0.015

Lymph nodes: Group I vs II *p* = 0.922, Group I vs III *p* = 0.028, Group II vs III *p* = 0.006

Liver resection extent: Group I vs II *p* = 0.002, Group I vs III *p* = 0.001, Group II vs III *p* = 0.001

Liver resection type: Group I vs II *p* = 0.016, Group I vs III *p* = 0.001, Group II vs III *p* = 0.001

Pringle indication: Group I vs II *p* = 0.625, Group I vs III *p* = 0.022, Group II vs III p = 0.01

Operation time: Group I vs II *p* = 0.05, Group I vs III *p* = 0.001, Group II vs III *p* = 0.001

Blood loss: Group I vs II *p* = 0.023, Group I vs III *p* = 0.001, Group II vs III *p* = 0.026

Blood transfusion: Group I vs II *p* = 0.001, Group I vs III *p* = 0.001, Group II vs III *p* = 0.006

Higher incidence of liver cirrhosis was found in Group I and II. Tumors were more commonly located in left hemi-liver in Group I and right hemi-liver in Groups II and III. Higher incidence of multiple tumors, macroscopic portal vein invasion, nearby organ invasion and presence of porta-hepatis lymph nodes were found in Group III. More major liver resections were performed in Group III. Pringle maneuver was more electively utilized in Groups I and II and emergently utilized in Group III. Longer operation time, more operative blood loss and more transfusion requirements were found in Group III.

### Postoperative data

Postoperative data of the study patients were summarized in Table 4. There were significant differences between the groups regarding ICU stay, total hospital stay, morbidities, posthepatectomy liver failure (PHLF), and respiratory complications. Longer hospital stay and more postoperative morbidities were noted in Group III. Also, more common posthepatectomy liver failure, and respiratory complications were noted in Group III.

**Table 4:** Post-operative data of the study patients (*ICU* Intensive care unit, *PHLF* Posthepatectomy liver failure, *US* Ultrasound, *ERCP* Endoscopic retrograde cholangio-pancreatography, *PVT* Portal vein thrombosis)

Variables	All (*N* = 315)	Group I (*N* = 88)	Group II (*N* = 167)	Group III (N = 60)	*P* value
ICU duration (days)	1 (1–22)	1 (1–6)	1 (1–22)	1 (1–7)	0.01
Hospital stay (days)	5 (2–66)	5 (5–22)	5 (2–31)	6 (3–66)	0.002
Morbidity	158 (50.2%)	36 (40.9%)	83 (49.7%)	39 (65%)	0.016
Clavien-Dindo grade					0.87
I	66 (21%)	16 (18.2%)	36 (21.6%)	14 (23.3%)	
II	47 (14.9%)	10 (11.4%)	25 (15%)	12 (20%)	
III-a	14 (4.4%)	3 (3.4%)	9 (5.4%)	2 (3.3%)	
III-b	11 (3.5%)	2 (2.3%)	5 (3%)	4 (6.7%)	
IV-a	2 (0.6%)	1 (1.1%)	0	1 (1.7%)	
V	18 (5.7%)	4 (4.5%)	8 (4.8%)	6 (10%)	
Morbidity type					0.064
General	4 (1.3%)	0	1 (0.6%)	3 (5%)	
Surgical	8 (2.5%)	2 (2.3%)	6 (3.6%)	0	
Liver-related	110 (34.9%)	26 (29.5%)	61 (36.5%)	23 (38.3%)	
Mixed	36 (11.4%)	8 (9.1%)	15 (9%)	13 (1.7%)	
PHLF	142 (45.1%)	32 (36.4%)	74 (44.3%)	36 (60%)	0.017
PHLF grade					0.643
A	81 (25.7%)	18 (20.5%)	46 (27.5%)	17 (28.3%)	
B	41 (13%)	10 (11.4%)	19 (11.4%)	12 (20%)	
C	20 (6.3%)	4 (4.5%)	9 (5.4%)	7 (11.7%)	
Bile leakage	17 (5.4%)	5 (5.7%)	8 (4.8%)	4 (6.7%)	0.851
Bile leakage treatment					0.255
Conservative	6 (1.9%)	2 (2.3%)	4 (2.4%)	0	
US guided tube	3 (1%)	1 (1.1%)	2 (1.2%)	0	
ERCP	7 (2.2%)	2 (2.3%)	2 (1.2%)	3 (5%)	
Operative	1 (0.3%)	0	0	1 (1.7%)	
Collection	16 (5.1%)	4 (4.5%)	7 (4.2%)	5 (8.3%)	0.44
Collection treatment					0.176
Conservative	6 (1.9%)	2 (2.3%)	1 (0.6%)	3 (5%)	
US guided tube	9 (2.9%)	2 (2.3%)	6 (3.6%)	1 (1.7%)	
Operative	1 (0.3%)	0	0	1 (1.7%)	
Internal hemorrhage Managed surgically	7 (2.2%)	1 (1.1%)	4 (2.4%)	2 (3.3%)	0.657
Wound infection All bed side management	9 (2.9%)	2 (2.3%)	4 (2.4%)	3 (5%)	0.541
Liver Abscess All US guided drainage	3 (1%)	2 (2.3%)	1 (0.6%)	0	0.298
Vascular complications (PVT)	5 (1.6%)	0	3 (1.8%)	2 (3.3%)	0.268
Respiratory complications	17 (5.4%)	5 (5.7%)	5 (3%)	7 (11.7%)	0.038
Pleural effusion	16 (5.1%)	5 (5.7%)	4 (2.4%)	7 (11.7%)	0.279
Pneumonia	1 (0.3%)	0	1 (0.6%)	0	
Respiratory treatment					0.189
Conservative	13 (4.1%)	3 (3.4%)	4 (2.4%)	6 (10%)	
US guided drainage	4 (1.3%)	2 (2.3%)	1 (0.6%)	1 (1.7%)	
Cardiac dysrhythmia	1 (0.3%)	0	0	1 (1.7%)	0.119
Renal complications (hepato-renal syndrome)	4 (1.3%)	2 (2.3%)	2 (1.2%)	0	0.476
Cerebral stroke	1 (0.3%)	0	1 (0.6%)	0	0.641
Ileus	1 (0.3%)	0	0	1 (1.7%)	0.119
Bleeding varices Endoscopic management	1 (0.3%)	0	1 (0.6%)	0	0.641
Early mortality	20 (6.3%)	4 (4.5%)	9 (5.4%)	7 (11.7%)	0.166
Early mortality cause Liver failure	20 (6.3%)	4 (4.5%)	9 (5.4%)	7 (11.7%)	0.166

ICU duration: Group I vs II *p* = 0.084, Group I vs III *p* = 0.007, Group II vs III *p* = 0.494

Hospital stay: Group I vs II *p* = 1, Group I vs III *p* = 0.002, Group II vs III *p* = 0.007

Morbidity: Group I vs II *p* = 0.182, Group I vs III *p* = 0.005, Group II vs III *p* = 0.05

PHLF: Group I vs II *p* = 0.232, Group I vs III *p* = 0.007, Group II vs III *p* = 0.05

Respiratory: Group I vs II *p* = 0.321, Group I vs III *p* = 0.227, Group II vs III *p* = 0.017

### Pathological data

Pathological data of the study patients were summarized in Table 5. There were significant differences between the groups regarding tumor size, number, resection margin, microvascular invasion, perineural invasion, tumor grade, and liver background. More multiple tumors, microvascular invasion, perineural invasion were found in Group III. More R0 resections were performed in Groups I and II. Higher incidence of pathologically confirmed liver cirrhosis was found in Group I and II.

**Table 5:** Pathological data of the study patients

Variables	All (*N* = 315)	Group I (*N* = 88)	Group II (*N* = 167)	Group III (*N* = 60)	*P* value
Size (cm)	6 (1.5–20)	4 (1.5–4.5)	6 (5–9.5)	11 (10–20)	0.001
Number					0.016
Single	272 (86.3%)	82 (93.2%)	144 (86.2%)	46 (76.7%)	
Multiple	43 (13.7%)	6 (6.8%)	23 (13.8%)	14 (23.3%)	
Resection margin					0.021
R0	275 (87.3%)	80 (90.9%)	149 (89.2%)	46 (76.7%)	
R1	40 (12.7%)	8 (9.1%)	18 (10.8%)	14 (23.3%)	
Capsular invasion	116 (36.8%)	36 (40.9%)	62 (37.1%)	18 (30%)	0.399
Microvascular invasion	147 (46.7%)	31 (35.2%)	74 (44.3%)	42 (70%)	0.001
Perineural invasion	122 (38.7%)	27 (30.7%)	61 (36.5%)	34 (56.7%)	0.004
Tumor Grade					0.002
I	57 (18.1%)	18 (20.5%)	33 (19.8%)	6 (10%)	
II	187 (59.4%)	55 (62.5%)	104 (62.3%)	28 (46.7%)	
III	61 (19.4%)	12 (13.6%)	28 (16.8%)	21 (35%)	
IV	9 (2.9%)	2 (2.3%)	2 (1.2%)	5 (8.3%)	
No viable tumor	1 (0.3%)	1 (1.1%)	0	0	
Liver background					0.04
Cirrhosis	298 (94.6%)	86 (97.7%)	159 (95.2%)	53 (88.3%)	
Normal	17 (5.4%)	2 (2.3%)	8 (4.8%)	7 (11.7%)	

Size: Group I vs II *p* = 0.001, Group I vs III *p* = 0.001, Group II vs III *p* = 0.001

Number: Group I vs II *p* = 0.145, Group I vs III *p* = 0.006, Group II vs III *p* = 0.103

Resection margin: Group I vs II *p* = 0.828, Group I vs III *p* = 0.02, Group II vs III *p* = 0.029

Capsular invasion: Group I vs II *p* = 0.589, Group I vs III *p* = 0.224, Group II vs III *p* = 0.348

Microvascular invasion: Group I vs II *p* = 0.182, Group I vs III *p* = 0.001, Group II vs III *p* = 0.001

Perineural invasion: Group I vs II *p* = 0.406, Group I vs III *p* = 0.002, Group II vs III *p* = 0.009

Tumor grade: Group I vs II *p* = 0.61, Group I vs III *p* = 0.005, Group II vs III *p* = 0.001

Tumor stage: Group I vs II *p* = 0.144, Group I vs III *p* = 0.001, Group II vs III *p* = 0.001

Liver background: Group I vs II *p* = 0.501, Group I vs III *p* = 0.031, Group II vs III *p* = 0.075

### Survival outcomes

#### Overall survival

**Fig. 1:**
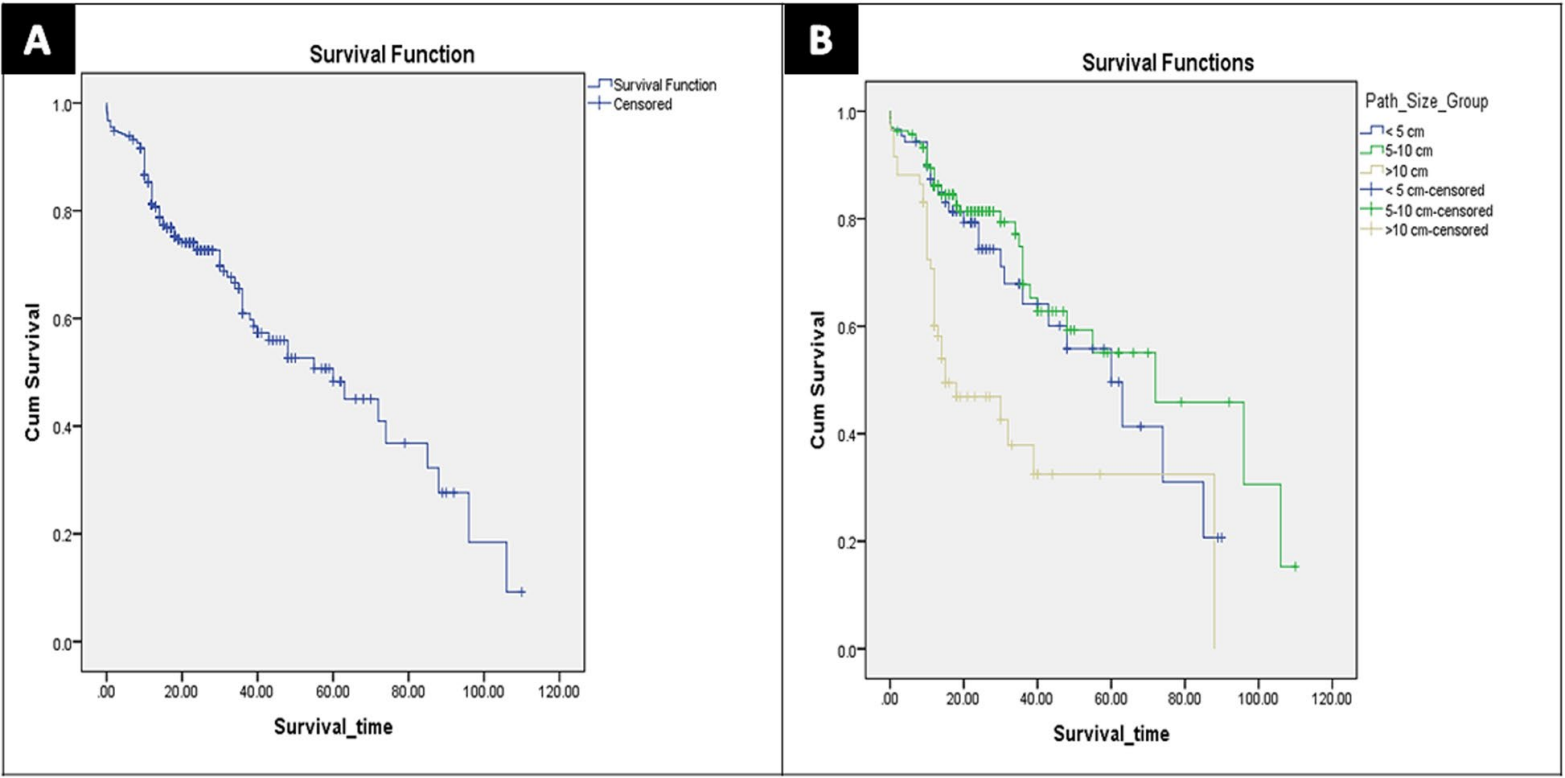
Kaplan Meier survival curves of all study cases. **A** Overall survival. **B** Disease-free survival

The median follow-up duration was 17 months (7–110 months). Mortality occurred in 100 patients (31.7%). The 1-, 3-, and 5-years OS rates of all study cases were 81.2%, 65.5%, and 48.3%, respectively (Fig. 1A).

**Fig. 2:**
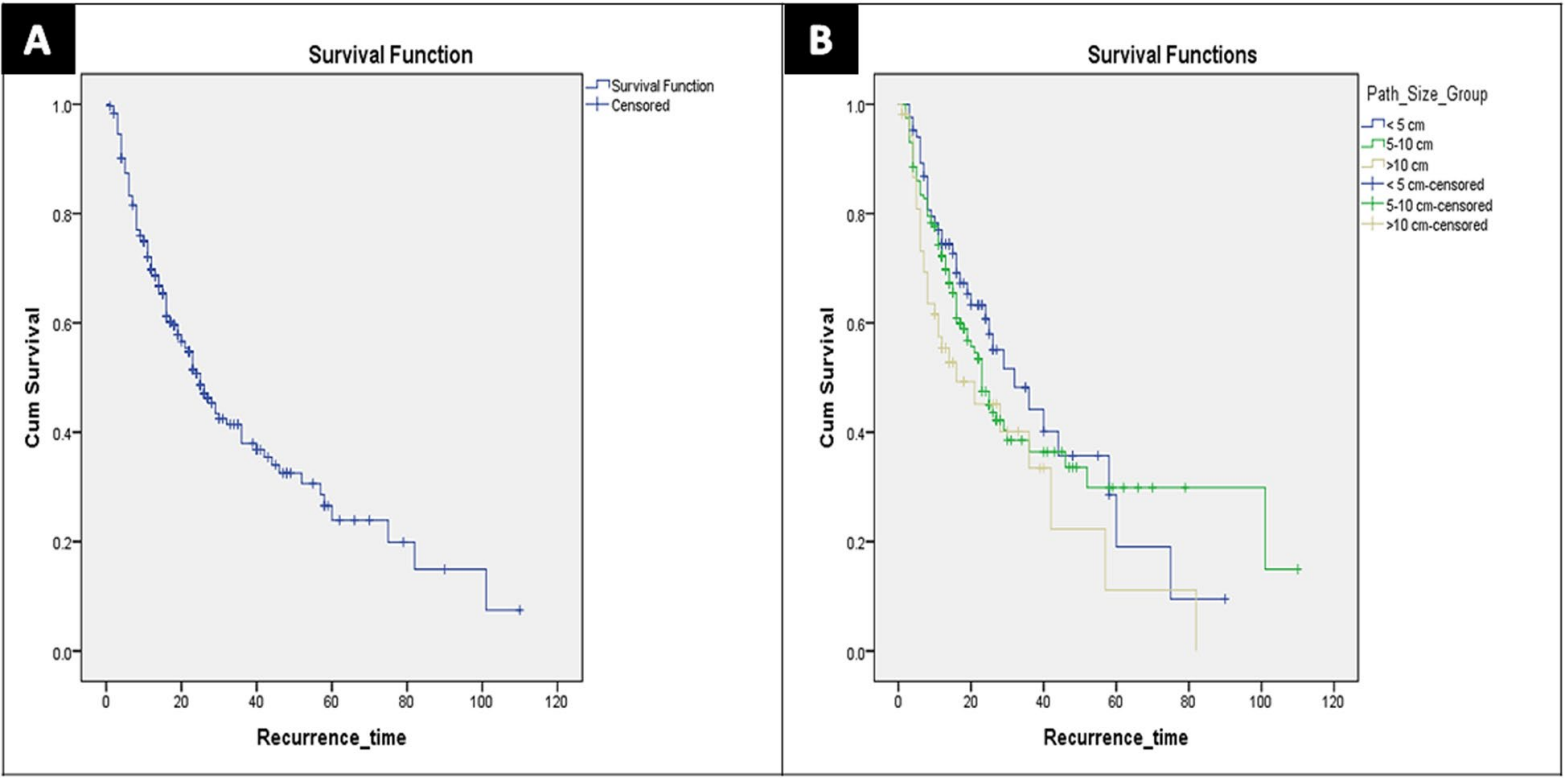
Kaplan–Meier survival curves comparing all study groups. **A** Overall survival (Log rank: Chi-Square = 27.4, df = 2, *p* = 0.001). **B** Disease-free survival (Log rank: Chi-Square = 4.8, df = 2, *p* = 0.089)

The 1-, 3-, and 5-years OS rates of Group I cases were 86.2%, 67.9%, and 49.6%, respectively. The 1-, 3-, and 5-years OS rates of Group II cases were 89.4%, 67.8%, and 55.1%, respectively. The 1-, 3-, and 5-years OS rates of Group III cases were 70.7%, 37.9%, and 32.5%, respectively (Log Rank: Chi-Square = 27.4, df = 2, *p* = 0.001) (Fig. 2A).

#### Disease-free survival

Recurrence occurred in 147 patients (46.7%). There were no significant differences between the groups regarding recurrence time, and site as shown in Table 6. The 1-, 3-, and 5-years DFS rates of all study cases were 72.1%, 41.4%, and 26.5%, respectively (Fig. 1B).

**Table 6:** Survival and recurrence data of the study patients (*TACE* Transarterial chemoembolization, *RFA* Radiofrequency ablation, *MWA* Microwave ablation)

Variables	All (*N* = 315)	Group I (*N* = 88)	Group II (*N* = 167)	Group III (*N* = 60)	*P* value
Mortality	100 (31.7%)	27 (30.7%)	40 (24%)	33 (55%)	0.001
Survival time (month)	17 (7–110)	17 (7–90)	18 (5–110)	13 (4–88)	0.036
Recurrence	147 (46.7%)	38 (43.2%)	78 (46.7%)	31 (51.7%)	0.376
Recurrence time (month)	14 (4–110)	15 (4–90)	14 (4–110)	12 (4–82)	0.053
Recurrence site					0.804
Intrahepatic	113 (35.9%)	30 (34.1%)	61 (36.5%)	22 (36.7%)	
Extrahepatic	6 (1.9%)	1 (1.1%)	4 (2.4%)	1 (1.7%)	
Both	28 (8.9%)	7 (8%)	13 (7.8%)	8 (13.3%)	
Intrahepatic site					0.059
Liver margin	7 (2.2%)	2 (2.3%)	2 (1.2%)	3 (5%)	
Same liver lobe	33 (10.5%)	12 (13.6%)	16 (9.6%)	5 (8.3%)	
Other liver lobe	44 (14%)	9 (10.2%)	20 (12%)	15 (25%)	
Bilobar	57 (18.1%)	14 (15.9%)	36 (21.6%)	7 (11.7%)	
Intrahepatic treatment					0.047
Resection	2 (0.6%)	1 (1.1%)	1 (0.6%)	0	
TACE	37 (11.7%)	6 (6.8%)	24 (14.4%)	7 (11.7%)	
RFA	9 (2.9%)	4 (4.5%)	4 (2.4%)	1 (1.7%)	
MWA	10 (3.2%)	5 (5.7%)	3 (1.8%)	2 (3.3%)	
Combined therapy	12 (3.8%)	7 (7.9%)	2 (1.2%)	3 (5%)	
Systemic therapy	1 (0.3%)	1 (1.1%)	0	0	
Supportive	70 (22.2%)	13 (14.8%)	40 (24%)	17 (28.3%)	
Extrahepatic site					0.492
Lung	13 (4.1%)	3 (3.4%)	5 (3%)	5 (8.3%)	
Bone	5 (1.6%)	1 (1.1%)	4 (2.4%)	0	
Brain	1 (0.3%)	0	1 (0.6%)	0	
Peritoneum	5 (1.6%)	2 (2.3%)	3 (1.8%)	0	
Adrenal gland	1 (0.3%)	0	0	1 (1.7%)	
Abdominal wall	1 (0.3%)	1 (1.1%)	0	0	
Lymph nodes	3 (1%)	1 (1.1%)	1 (0.6%)	1 (1.7%)	
Multi-site	5 (1.6%)	0	3 (1.8%)	2 (3.3%)	

Mortality: Group I vs II *p* = 0.298, Group I vs III *p* = 0.003, Group II vs III *p* = 0.001

Intrahepatic recurrence treatment: Group I vs II *p* = 0.014, Group I vs III *p* = 0.301, Group II vs III *p* = 0.485

The 1-, 3-, and 5-years DFS rates of Group I cases were 77%, 48.2%, and 28.6%, respectively. The 1-, 3-, and 5-years DFS rates of Group II cases were 74.2%, 38.5%, and 29.9%, respectively. The 1-, 3-, and 5-years DFS rates of Group III cases were 57.5%, 33.5%, and 11.2%, respectively (Log Rank: Chi-Square = 4.8, df = 2, *p* = 0.089) (Fig. 2B).

## Discussion

HCC is one of the most prevalent neoplasms all over the world. HCC is one of the most lethal neoplasms worldwide and it represents the second most common cause of tumor associated mortalities [21, 22]. The condition is not much different in Egypt. The Egyptian Health Authorities considered HCC as the most challenging health care problem among Egyptians. The prevalence of HCC is continuously rising among Egyptian patients owing to the high prevalence of hepatitis C viral (HCV) infection (genotype 4) [23].

Liver resection is one of the curative lines of HCC patients especially in patients with early HCC with well-preserved liver functions with acceptable perioperative and long-term outcomes [24, 25]. On the other hand, the presence of liver cirrhosis may lead to higher incidence of perioperative morbidity and mortality with poorer long-term survival outcomes [26]. The current study was conducted to review our center experience of liver resection for HCC and evaluate the prognostic impact of tumor size on the outcomes of liver resection for HCC among Egyptian patients, in area where hepatitis C virus (genotype 4) is the main predisposing factor for HCC development.

Tumor size is an important predictive factor for the outcomes after surgical treatment for HCC. This might be attributed to the fact that larger HCCs are frequently associated with some adverse prognostic factors like the presence of microscopic vascular invasion. Also, it is known that the prognosis of patients undergoing curative resection for larger HCC is inferior to those with small HCC [10, 11]. Therefore, tumor size should be considered as one of the most important predictive factors for tumor recurrence and patient survival [4, 9]. In general practice, there is no size limitation regarding liver resection for HCC if the remnant liver volume is adequate. This is not clearly matched with the recent practice guidelines of BCLC staging system due to the great variations between areas in social, environmental, and medical conditions [27].

Liver resection especially in cirrhotic patients is always associated with more blood loss and higher needs for perioperative transfusions. In the current study, pathological liver cirrhosis was found in 298 patients (94.6%). Previous studies had addressed that blood loss and perioperative transfusions are associated with the development of immunological reactions and nonspecific immunosuppression, which subsequently affect the development of postoperative morbidities and negatively affects the prognosis of HCC patients [28, 29]. In the current study, we noticed longer operation time, more intraoperative blood loss and more transfusion requirements among patients with larger tumors compared to counters with smaller ones. This is related to the more frequent major liver resections and more macroscopic portal vein invasion (PVI) among this group of patients.

HCC has a high affinity for macroscopic portal vein invasion (PVI). The presence of macroscopic PVI is considered a strong negative prognostic factor for HCC patients. This is attributed to the high risk of dissemination of the cancer cells into the blood stream and metastasis to to other parts of the liver and distant other organs [30, 31]. We previously reported that surgical management of selected HCC patients associated with the presence of macroscopic PVI is technically feasible and is associated with comparable recurrence free survival but poorer overall survival, when compared to a matched group of HCC patients without macroscopic PVI [14]. In the current study we found significantly higher incidence of macroscopic PVI among patients with large sized HCCs compared to smaller HCCs, especially in patients with tumors larger than 10 cm. This is denoting a strong correlation between tumor size and the presence of macroscopic vascular invasion.

In the current study, we found a higher incidence of post-operative morbidities in HCC patients with larger tumors, especially the incidence of PHLF and respiratory complications. Previous studies had shown a varying incidence of postoperative morbidities after liver resection for HCC reaching up to 70%. Differences in the incidence of post-operative morbidities are mainly related to the differences in the patient selection and background liver disease. The commonest complications encountered were postoperative bleeding, liver dysfunction or liver failure, ascites, and sepsis [32–36]. In previous studies from our center, we reported that liver resection among patients with HCV-related HCC is associated with high incidence of perioperative morbidities. We also, reported a higher incidence of PHLF among Egyptian patients with HCV-related HCC compared to other studies [5, 8].

Tumor recurrence after liver resection for HCC negatively affects the prognosis of HCC patients. Tumor recurrence may occur early after resection owing to multicentric carcinogenesis or late after resection because of the presence of coexisting background of liver cirrhosis [37]. Several factors had been identified to affect tumor recurrence which could be classified to tumor-related, procedure-related, and patient-related factors [38–40]. Lurje et al. in a study evaluating tumor recurrence and patients’ survival after curative liver resection for HCC addressed that tumors within Milan criteria, macrovascular invasion, and tumor stage were independently associated with recurrence, while macrovascular invasion and MELD score were independently associated with survival [40]. Wang et al. in a study evaluating the long-term outcomes after liver resection for HCC addressed that in patients with Ishak stage 1 to 5, tumor size was associated with postoperative mortality, and tumor size, and AFP > 20 ng/ml were associated with recurrence rate. On the other hand, poorly differentiated histology and tumor size were associated with higher mortality, and tumor size was associated with recurrence in patients with Ishak stage 6 [41]. Dai et al. in a study evaluating the impact of tumor size on the prognosis of HCC in patients who underwent liver resection showed the important role of tumor size and advanced fibrosis in predicting postoperative mortality and the role of tumor size and histopathological differentiation in predicting HCC recurrence [42]. In the current study, the survival outcomes of the study patients were clearly stratified according to the tumor size. Patients with smaller tumors showed better survival outcomes while patients with larger tumors experienced worser survival outcomes. There were significant differences between the groups regarding the overall survival but not the disease-free survival.

The current study has some limitations. This is a retrospective, single-center study which is liable to selection bias. Secondly, we included patients with HCCs on top of hepatitis C virus, which is the commonest risk factor among Egyptian patients. On the other hand, we did not include any modality of artificial intelligence or machine learning in the current analysis which is one of the most critical advancements in the recent years and is growing in popularity for analysis of large amounts of data. A future multicenter prospective study is needed to validate our results.

## Conclusion

In conclusion, the current study suggested that the tumor size is not a contraindication for liver resection. Patients with larger tumors showed higher incidence of perioperative morbidities, especially PHLF and respiratory complications, but comparable peroperative mortality. Patients with larger tumors showed comparable recurrence free survival but poorer overall survival when compared to their counters with smaller tumors.

## Data Availability

The data generated and analyzed for the current manuscript is not publicly available and will be available by a reasonable request from the corresponding author.
